# The Impact of Innovative Smoking Reduction Education at Hospital Entrances: A Prospective Pre- and Post-Test Study Design

**DOI:** 10.3390/ijerph15091922

**Published:** 2018-09-04

**Authors:** Tusi-Ping Chu, Min-Li Chen, Yu-Chen Lin, Mei-Yen Chen

**Affiliations:** 1 Department of Nursing, Chang Gung Memorial Hospital, Chiayi 613, Taiwan; E57879@cgmh.org.tw (T.-P.C.); mlchen@gw.cgust.edu.tw (M.-L.C.); 2 Department of Nursing, Chang Gung University of Science and Technology, Chiayi 613, Taiwan; cyhd235@mail.cyshb.gov.tw; 3 Department of Respiratory Care, Chang Gung University of Science and Technology, Chiayi 613, Taiwan; 4 Department of Health Promotion, Chiayi Bureau of Health, Chiayi 613, Taiwan; 5 Department of Nursing, Chang Gung University, Taoyuan 333, Taiwan; 6 Department of Cardiology, Chang Gung Memorial Hospital, Yunlin 638, Taiwan

**Keywords:** smoking cessation education, nursing students, nursing education, hospital entrance

## Abstract

*Background:* Nurses are expected to play an important role in smoking reduction education. Although the smoking ban was enacted in Taiwan in 1997, which included the introduction of smoking-free areas around the entrances of hospitals, many smokers are still found near hospitals. Few smoking reduction programs have been initiated around hospital entrances. The aim of this study was to examine the impacts of an innovative smoking reduction education program, which was conducted by nursing students around the entrances of a teaching hospital. *Methods:* A prospective pre- and post-test study design was used. The smoking reduction education program consisted of posters, audio broadcasts, and dramatic performances that provided information and resources related to smoking reduction. Outcome variables included the number of smokers, number of cigarette butts on the ground, and the experiences from nursing students after conducting the program. *Results:* After adjusting for weather and temperature, the number of smokers decreased significantly in the afternoon and during the whole day after the implementation of the program. The number of cigarette butts significantly decreased during the implementation of the program. *Conclusion:* The findings reveal that alternative smoking reduction programs initiated around hospital entrances significantly reduced both the number of smokers and cigarette butts on the ground. Nursing students shared their positive experiences in conducting this study.

## 1. Introduction

Ideally, hospitals should promote a healthy environment. Ironically, many smokers stand outside hospital entrances. This juxtaposition of smokers and hospitals may reduce the impact of smoking reduction education. Recently, Taiwan officially reported that cigarette smoking has significantly declined in adults, from 32.5% in 1990 to 15.3% in 2016 [1]. Excluding the low prevalence rate in women, the smoking rate for men in Taiwan was 28.5% in 2016 [1]. In 1997, Taiwan enacted a smoking ban, which was amended in 2009, that prohibits smoking in indoor areas of public places and of organizations’ properties, such as the entrances of hospitals [2]. In addition, Taiwan had established the first Global Network for Tobacco Free Healthcare Services (GNTH) around the Asian Pacific region in 2011 [2]. In this network, providing smoke-free environments and smoke reduction policies are recognized as an important responsibility for hospitals, including healthcare providers, inpatients, and visitors [2]. Unfortunately, it is still very common for people to smoke around the entrances of hospitals, even teaching hospitals. Until 2017, in total, only 11 hospitals received the certification by the GNTH nationwide [2]. Therefore, it is necessary to reduce the problem of smoking in front of hospitals by launching antismoking action.

Cigarette smoking is one of the main and preventable risk factors for a number of chronic diseases, including lung disease, oral disease, many types of cancer, and cardiovascular disease, which is caused by vascular disruption, inflammation, endothelial dysfunction, and increased vascular stiffness [3,4]. Many studies indicate that active and persistent smoking can worsen the degree of disabilities and disease clusters, including beta cell dysfunction, increased visceral fat, insulin resistance, metabolic syndrome, carotid plaque formation, and diabetes mellitus [5,6].

Tobacco kills more than seven million people each year worldwide. More than six million of those deaths are the direct result of tobacco use, and nearly one million are the result of nonsmokers being exposed to secondhand smoke [1]. There are more than 4000 chemicals in tobacco smoke, of which at least 250 are known to be harmful and more than 50 are known to cause cancer [1]. Each year, the World Health Organization [1] publishes a “World No Tobacco Day” brochure, which addresses how tobacco control promotes sustainable development and promotes legislation restricting tobacco advertising and regulating who can buy and use tobacco products, as well as limiting where people can smoke. In light of tobacco’s addictive properties, many nurse-led counselors perceive barriers to the implementation of effective smoking reduction programs [7].

Many studies indicate that clinicians should offer and provide effective smoking reduction interventions to their smoking patients. Nurses are expected to play an important role in smoking reduction education because they are the largest group of healthcare providers [8,9,10,11]. Interestingly, some studies have indicated that the prevalence of tobacco use among nurses and nursing students is very high in some countries [12,13,14]. This phenomenon might impact nurses’ attitudes toward addressing smoking reduction with their patients. However, from a public health perspective, nurses are considered to be an important group for delivering smoking reduction interventions [13]. Three decades ago, Clark [15] mentioned that the nursing curriculum should help nurses develop their health educational role in smoking reduction.

Previous studies indicate that most of the nurse-led smoking reduction settings are conducted in hospital inpatient and outpatient clinics and in community-based screening settings [7,9,16]. In addition, many of the smoking reduction education strategies consisted of traditional instruction, pamphlets, minifilms, online education, and mobile technology with a short message service [17,18,19,20]. No research had focused on smokers at hospital entrances. Therefore, the purpose of this study is (1) to examine the impacts of an innovative model of a smoking reduction education program around two major entrances of a teaching hospital, and (2) to explore the experiences from nursing students after conducting the program. This program was guided by the health belief model, which was originally proposed by Rosenstock and subsequently modified by Becker [21]. The health belief model is based on the assumption that people’s preventive health behaviors are modified by their beliefs as to the severity of the disease and cues to action. The health belief model suggests that whether people could successfully change their behavior will be influenced by the cues from their relatives or healthcare personnel [21]. For instance, putting smoke-free signs or using audio to broadcast no-smoking information around outdoor areas of the hospital might enhance the cues for people to stop the habit. Therefore, this smoking reduction education program consisted of relaying important information related to the cues to reduce or stop smoking.

## 2. Materials and Methods

This study was conducted around two major entrances of a teaching hospital with 500 beds located in southern Taiwan. A prospective pre- and post-test study design was used.

### 2.1. Procedures and Data Collection

The data collection procedure was divided into three phases: phase 1, baseline data collection before intervention; phase 2, data collection combined with intervention (during intervention); and phase 3, after intervention. In total, we collected data for 75 days between April and September in 2017: phase 1: 24 days, phase 2: 30 days, and phase 3: 21 days.

Preparation phase (5 April–4 June): During the preparation phase, we conducted the following five steps: (1) collaborated with the Chiayi Bureau of Health Promotion, for its financial and legislative research support; (2) discussed the project with hospital administration, including security, to ensure protection of the nursing students during the project; (3) established a data collection form and method after discussion with experts, including how to record the number of cigarette butts and number of smokers; (4) educated the third-year group of 27 nursing students on smoking reduction education and developed the content of the program; and (5) conducted a pilot study and modified the observation and implementation time. The contents of the smoking reduction program included reviewing the hazards of cigarette smoking, understanding human behavior, and creating an innovative design for smoking reduction strategies based on the health belief model. Our selected trainers included research investigators, a professor in health communication, and the director of the Tainan Sweet Children Theater. Through an interdisciplinary collaboration, nursing students learned how to create (a) seven smoking reduction posters (100 × 75 cm) with meaningful information and slogans, (b) two versions of audio recordings (2–3 min, repeated 5–7 times daily when walking around the entrances) to replay the significant information related to smoking reduction and resources for smoking clinics and websites, and (c) a situational drama with 6 students (8–10 min) and a quit-smoking dance (around 5 min) to communicate positive energy.

Phase 1 (5 June–29 June): Using the form we developed during the preparation phase, we measured the number of smokers and other factors. We recorded data at 10 periods, using groups of two students to observe and record the information (including the weather, temperature, number of cigarette butts, and number of smokers) on the form, hourly between 8:00 a.m. and 5:00 p.m. every day. Phase 2 (30 June–4 August): Students performed the smoking reduction education program at the entrance of the hospital. In addition to recording the number of smokers and cigarette butts hourly, they carried smoking reduction posters and played the audio broadcast records from 8:00 a.m. to 5:00 p.m. Additionally, during three hours of heavy smoking between 11:00 a.m. and 4:00 p.m., six students performed the situational drama together with the quit-smoking dance, which lasted approximately 20 min, at two major hospital entrances. Phase 3 (8 August–11 September): During this phase, the nursing students continued counting and recording the number of smokers and cigarette butts between 8:00 a.m. and 4:00 p.m., with gradual reduction of the duration of the situational drama and quit-smoking dance.

### 2.2. Ethical Considerations

Prior to conducting this study, the institutional review board of Chang Gung Memorial Hospital (IRB No: 201700685B1) approved the conduct of this study.

### 2.3. Measurements

We considered that the weather and temperature may affect the number of people smoking outside the hospital. For instance, on rainy days, smokers may not go outside, and this may cause errors in measurement. Accordingly, the weather and temperature were measured as potential confounders and recorded using the following parameters on the form:

Weather and temperature: Based on the report from the Central Weather website, the weather and temperature were recorded in the morning and afternoon during phases 1, 2, and 3, and the weather was classified as sunny, cloudy, or rainy.

Cigarette butts and smokers: The hospital cleaner cleaned the cigarette butts on the grounds every hour from 6:00 a.m. to 6:00 p.m. Thus, a group of two students observed and recorded the gender of smokers and cigarette butts at 10 locations around two major entrances of hospital every hour (8:00 a.m.–5:00 p.m.). After they counted the cigarette butts, the hospital cleaners promptly cleaned the ground. The pattern was repeated hourly.

Subjective experiences: After conducting the program, students were encouraged to answer an open-ended question: how do you feel about conducting this project? The content analysis was used.

### 2.4. Data Analysis

The weather during the three phases—before intervention (phase 1), during intervention (phase 2), and after intervention (phase 3)—was compared using Fisher’s exact test. Continuous data (temperature) during the three phases was compared using one-way analysis of variance. Multiple comparisons between any two phases were made using the Bonferroni adjustment of probability. The intervention effect after the program was tested using multivariable linear regression analysis, in which weather and temperature were adjusted. The estimated marginal mean (adjusted mean) in each phase was obtained and compared using the Bonferroni adjustment of probability. All tests were two-tailed and *p* < 0.05 was considered statistically significant. Data analysis was conducted using SPSS 22 (IBM SPSS Inc., Chicago, IL, USA).

## 3. Results

### 3.1. Descriptive Analysis

The weather status and temperature among three phases were measured. In the morning, there was no difference in the weather between the three phases. However, the temperature in the mornings was higher than in phase 2 (32.5 ± 1.9) and phase 3 (35.3 ± 1.8; *p* < 0.001). In the afternoon, the percentage of sunny days in phase 2 (26, 86.7%) and phase 3 (17, 81%) was much higher than in phase 1 (10, 41.7%; *p* = 0.001). Similar to the mornings, the temperature was the highest in phase 3 (35.9 ± 1.8), followed by phase 2 (34.3 ± 2.0) and then phase 1 (31.5 ± 3.3).

### 3.2. Effect of Smoking Reduction Education

**Figure 1:**
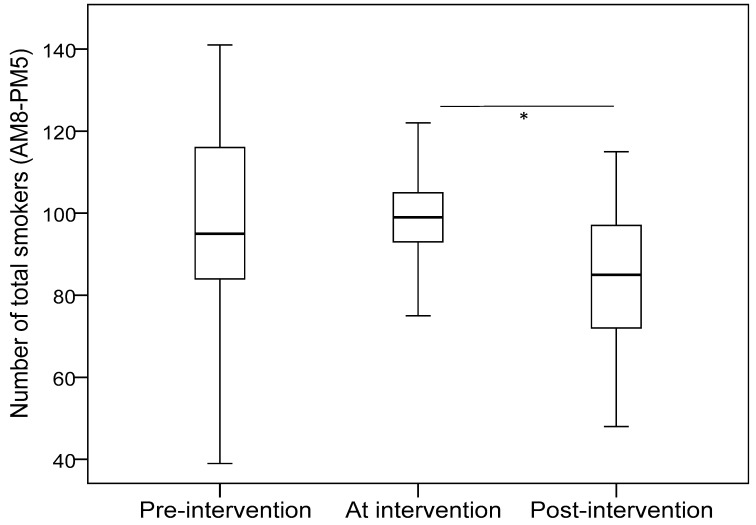
Number of total smokers for the whole day among the three phases of the study. * indicates *p* < 0.05.

**Figure 2:**
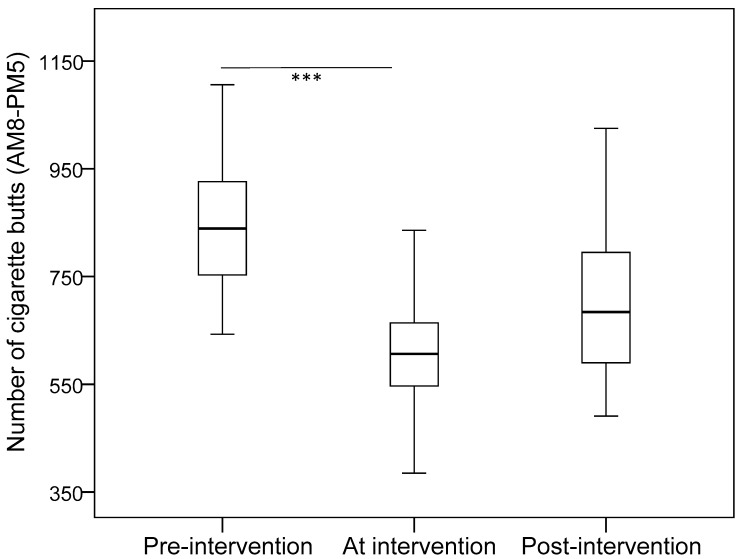
Number of cigarette butts during the whole day among the three phases of the study. *** indicates *p* < 0.001.

Table 1 shows the estimated mean in each phase after adjusting for weather and temperature. In the mornings, the number of cigarette butts significantly decreased in phase 2 (compared with phase 1), but significantly increased in phase 3 (compared with phase 2). In the afternoon, the number of male smokers and total smokers decreased in phase 3 as compared with phase 1 and phase 2. The number of cigarette butts was less in phase 2 and phase 3 than phase 1. When adding the data from the morning and afternoon, the results indicated that the number of male smokers and total smokers decreased in phase 3 as compared with phase 2. The number of cigarette butts significantly decreased between phase 1 and phase 2, but there was no difference between phase 1 and phase 3 (Bonferroni multiple comparison: *p* = 0.176). Figure 1 and Figure 2 illustrate the median values of total smokers and cigarette butts for the whole day in the three phases of the study.

### 3.3. Subjective Responses from Nursing Students

After the end of the five-month project, students were encouraged to write an answer to an open-ended question: how do you feel about conducting this project? All of the students’ responses are provided below, although some are only quoted in part:

*The image that most impressed me... was a mother smoking in front of her sick baby, which terrified me extremely... I wanted to do my best efforts to distribute the reduction education program to eliminate the smoking environment.*

*When seeing us approach, the smokers would hide their cigarettes or silently hide from us. Though they didn’t physically quit smoking immediately, we were still pleased to realize that friendly persuasion could somehow impact those smokers.*

*A primary school girl happily took her cell phone to record our situational drama and quit smoking dance because she wanted to persuade her smoking Dad and Grandpa at home with our inspired performance. How touching the little sweet girl was!*

*“Could we tape the live show of your performance? It is interesting and vigorous. We want to share it with our IG fans.” One young patient’s family praised our novel mission and gave us emotional supports.*

*“You are good guys to do such meaningful things! Keep going on! We certainly back you up all the time.” A tender Grandma smiled to encourage us and gave us a marvelous gesture.*

*Three male smoking patients ridiculed each other when seeing our situational drama performance. “Hey, you should drop your cigarette butts! Don’t you see that smoking harms you?” “You are the biggest addict among us, aren’t you? You just behave yourself, don’t tease others!” “Fine, since we don’t want to become the victims of smoking, let’s try our best to quit smoking together!” Their inspirational conversation to us, no matter how hard, was deserved.*

*In the hot summer days, we have to record the smokers and butts under the burning sunshine... per hour. We felt exhausted with stinky sweat... but the general public’s support, encouragement, and positive praise would cheer us up to keep working harder and harder. What meaningful work.*

*I think the innovative situational drama performance is significantly positive. … We discovered the number of smokers was becoming less and less during our project.*

*One day, I was astonished by a middle-aged uncle who went straight to me with a lit cigarette in hand. Instead of scolding or blaming me, he suddenly asked me to extinguish his cigarette and throw it away at once. He declared to quit smoking. How impressive to us!*

*I never thought that we can do some different ways of smoking reduction education... like this. We can try alternative ways different from traditional ways. This project caused me to think or adopt new solutions in behavioral change. It is so interesting to me, I never had this experience of conducting smoking reduction.*

## 4. Discussion

The aim of this study was to explore the impacts of an alternative method of smoking reduction education around the entrances of a teaching hospital. Although some limitations exist in this study, the results revealed significant implications in terms of the nursing curriculum and smoking reduction education policies. Three key findings emerged from this study: First, the number of smokers decreased significantly in phase 3 in the afternoon, as well as during the whole day. Second, the number of cigarette butts significantly decreased in phase 2 during all three periods of the day. Third, nursing students shared their positive experiences from during the conduction of the project, which led us to recommend including creative ways of implementing smoking reduction programs in the nursing education curriculum.

Although smoking is prohibited by law at the entrances of hospitals [2,22], it is difficult to comprehensively enforce the ban because the resources of the police and the district inspector are limited. Although the present study indicated that the smoking reduction education program significantly decreased the number of smokers and cigarette butts thrown on the ground during phases 2 and 3, the average number of smokers per day is still high. For instance, in phase 3, an average of 78 people per day smoked outside the hospital entrances. Moreover, we believe this value is less than the actual number of smokers. Despite the above findings, we also found that the intervention failed in the morning period. The possible reasons might be due to the weather; since the temperature is lower than in the afternoon (the average temperature was 34–35 °C), the smokers tended to go outside in the morning. In addition, this study was found to have a Hawthorne effect [23], which is a type of reactivity whereby individuals modify an aspect of their behavior in response to their awareness of being observed. The result showed the number of smokers and cigarette butts decreased in phase 2, but increased again in phase 3.

The present finding was similar to a study in Australia. Nagle et al. [24] conducted an observation study to describe the type and location of smokers on the smoke-free grounds of public hospitals and to observe the impact of introducing smoke-free signs before and after the intervention at one hospital. The results indicated a slightly decreasing rate of smokers around less than 10 m from entrances to the hospital building. They also pointed out that among all smokers, less than 10% of observed outdoor smokers were patients, 40% were visitors, and more than 50% were hospital staff. Furthermore, Poder et al. [25] initiated a two-year study to evaluate the compliance of hospital staff, inpatients, and visitors with smoke-free environment policy. After the implementation, they found the smoking incidents were reduced by 44% (*p* ≤ 0.05) in staff, 37% (*p* ≤ 0.05) in visitors, and remained unchanged among inpatients. Their studies inspired us that conducting a smoke-free strategy around hospital was effective and worthwhile to do. Unlike the above studies, we do not know how many of the smokers we counted were different people (or were just the same smokers smoking repeatedly throughout the day) and we know nothing about the smokers’ background (e.g., patients, visitors, or hospital employees). Therefore, it is necessary to conduct further studies to capture the additional information (e.g., accompanied by an inspector).

To ensure a better future, we need a comprehensive tobacco policy. In a study in the United States, Weaver et al. [26] compared the effects of a tobacco policy before and after initiation in Indianapolis and Marion County in Indiana, and found that it significantly decreased the admission rate of acute myocardial infarction in five Marion County hospitals. According to a systematic review, Palmer et al. [17] concluded that smoking cessation delivered by mobile technology with a short message service increases quitting rates. This finding inspires us to seek new ideas using technology or artificial intelligence, such as when visitors are near a hospital, an application program could automatically initiate the audio broadcast recording with smoking reduction education content.

Unlike the high smoking prevalence for health personnel and nursing students in Western countries (22–44%) [12,13,14,27], the smoking rates for women, healthcare personnel, and nursing students in Taiwan are very low (2–4%) [8]. In this study, all of the nursing students reported that they are not current or former smokers. Although this is an asset for the healthcare delivery system in Taiwan, we worry that many nursing students do not have any experience with smoking reduction education in their curriculum or practicum. As many nursing scholars have stated [10,11,18,19], nurses form the largest cohort of healthcare professionals. Thus, nurses should play an important role in smoking reduction because they are influential in modifying patient behaviors. The literature indicates that effective smoking cessation/reduction practices include using medication or individualized behavior modification to quit smoking [1,2]. However, few curriculums designed had included the innovative smoking reduction strategies to monitor the compliance of a smoke-free environment policy in a healthcare setting. From the subjective responses by nursing students regarding conducting this project, we learned also from their positive experiences, such as: “*It is so interesting to me, I never had this experience…from general public’s support and encouragement through their performance.*” In addition, nursing curriculums need to emphasize the role that nurses play in smoking reduction and empower them with the tools, knowledge, and skills they need to help patients quit or reduce cigarette use and to prevent nurses from smoking within the general population [10].

Smoke-free environment policies have been developed and introduced worldwide [1,2]. These policies reduce tobacco use and protect the community from environmental tobacco smoke. Therefore, how to encourage people not to smoke in certain areas is an important mission for the managers of hospital and policy makers. The major strength of this study is that it focuses on a neglected area in a healthcare setting where many smokers gather. The results indicate that policymakers should consider ways to enhance hospitals’ positive leadership roles in communities, particularly mission-driven teaching hospitals. This study also highlighted the pivotal role of smoking reduction education in the nursing curriculum. 

Despite the valuable findings in this study, some limitations should be noted. First, the nonrandom sampling of one hospital and the limited geographical scope limited the generalizability of these findings. Second, based on the absence of a control group, potential threats to the internal validity of the program must be considered. Third, measurement errors may have occurred, because the number of smokers and cigarette butts were counted only once without confirmation through a crosscheck review. Fourth, due to the short period of observation, we do not know if the applied smoking reduction education could physically change smokers’ behaviors. Furthermore, the values may underestimate the number of smokers and cigarette butts because only people actively smoking were counted, and not people who were standing with a cigarette in hand.

## 5. Conclusions

The smoking reduction education program appears to be an intervention that can be implemented as a means of alternative innovation at hospital entrances. Although this study has its limitations, nursing students reported positive outcomes in conducting an alternative smoking reduction education program which was supervised by interdisciplinary experts. The smoking reduction education program’s effects on decreasing the number of smokers and cigarette butts should be evaluated in a future study with longer follow-up periods.

## Figures and Tables

**Table 1:** Effect of smoking reduction education on the number of smokers and cigarette butts outside of the target hospital.

Dependent Variable	Estimated (Adjusted) Mean ± Standard Error ^‡^		
	Phase 1	Phase 2	Phase 3
Morning (08:00–12:00)			
Number of male smokers	41.2 ± 4.8	42.1 ± 3.7	42.1 ± 3.7
Number of female smokers	5.3 ± 1.1	4.7 ± 0.8	3.7 ± 0.8
Number of total smokers	46.8 ± 5.2	47.0 ± 4.0	44.8 ± 4.0
Number of cigarette butts	407.5 ± 42.4	289.0 ± 32.7 ^a^	367.8 ± 32.4 ^b^
Afternoon (13:00–17:00)			
Number of male smokers	43.7 ± 4.9	44.4 ± 3.8	29.2 ± 3.7 ^a,b^
Number of female smokers	4.9 ± 0.9	4.6 ± 0.7	4.4 ± 0.7
Number of total smokers	48.3 ± 5.4	48.8 ± 4.2	33.6 ± 4.1 ^a,b^
Number of cigarette butts	424.2 ± 37.2	294.3 ± 28.7 ^a^	319.7 ± 28.5 ^a^
Whole day (08:00–17:00)			
Number of male smokers	84.9 ± 8.1	86.5 ± 6.2	71.4 ± 6.2 ^b^
Number of female smokers	10.2 ± 1.3	9.3 ± 1.0	8.0 ± 1.0
Number of total smokers	95.1 ± 8.8	95.7 ± 6.8	78.3 ± 6.7 ^b^
Number of cigarette butts	831.7 ± 65.7	583.4 ± 50.7 ^a^	687.5 ± 50.3

^‡^ Adjusted for weather and temperature; ^a^ indicates *p* < 0.05 vs. phase 1 in the Bonferroni multiple comparisons; ^b^ indicates *p* < 0.05 vs. phase 2 in the Bonferroni multiple comparisons.
